# Effectiveness of ACTH in Patients with Infantile Spasms

**DOI:** 10.3390/brainsci12020254

**Published:** 2022-02-11

**Authors:** Justyna Paprocka, Jakub Malkiewicz, Veronica Palazzo-Michalska, Barbara Nowacka, Mikołaj Kuźniak, Ilona Kopyta

**Affiliations:** 1 Department of Pediatric Neurology, Faculty of Medical Sciences in Katowice, Medical University of Silesia, 40-752 Katowice, Poland; ilonakopyta@autograf.pl; 2 Department of Neurology, Faculty of Medical Sciences in Katowice, Medical University of Silesia, 40-752 Katowice, Poland; jjmalkiewicz@onet.pl; 3 Students’ Scientific Society, Department of Pediatric Neurology, Faculty of Medical Sciences in Katowice, Medical University of Silesia, 40-752 Katowice, Poland; ver.palazzo@gmail.com (V.P.-M.); b.m.w.nowacka@gmail.com (B.N.); mikolajkuz@wp.pl (M.K.)

**Keywords:** West syndrome, infantile spasms, treatment, ACTH, tetracosactide, vigabatrin

## Abstract

(1) Background: West syndrome is a severe, refractory, epileptic syndrome that usually appears in infancy or early childhood. ACTH is one of the more effective drugs for treating this condition. (2) Aim of the study and methods: The objective of our study was to examine short-term efficacy (during treatment schedule) and long-term outcome of intramuscular 0.02 mg/kg/day ACTH (tetracosactide) depot, used concomitantly with other antiepileptic drugs (AEDs) in patients with infantile spasms who did not achieve seizure cessation or relapse when taking only the AEDs. The drug efficacy was evaluated in retrospective and prospective analyses of 50 patients diagnosed with infantile spasms. (3) Results: Complete cessation of spasms was achieved in 42 cases (84%). EEG improvement was seen in 41 (82%) patients who responded to ACTH therapy. Information on the clinical course of 28 patients was obtained duringlong-term follow-up. In 17 (60.7%) cases, seizures were still present. Normal or near-normal development was observed in 11 out of 28 children (39%). ACTH used concomitantly with other AEDis a highly effective treatment with acceptable side effects. (4) Conclusion: Randomized controlled clinical trialswith long-term follow-up are needed to compare the effectiveness of ACTH in polytherapy and monotherapy. Dyskinesias as a potential side effect observed in our study group should be investigated in the following studies.

## 1. Introduction

Infantile spasms (IS) is a challenging epileptic syndrome to treat. Its incidence is approximately from 1:4000 to 1:6000 live births [1], constituting 13–14.5% of all cases of infantile epilepsies [2]. It can also be called infantile epileptic encephalopathy since the continuous, disorganized, and arrhythmic electrical activity of neuronal networks may influence brain maturation, cause arrest or regression of previously gained functions, or make infant milestones impossible to achieve [2]. Infantile spasms consist of three features: epileptic spasms, developmental arrest or regression, and hypsarrhythmia—an interictal EEG pattern. However, not all of them must be present. Hypsarrhythmia together with epileptic spasms in clusters are obligatory features to diagnose West syndrome (WS), according to the West Delphi Proposal, but some studies use the terms West syndrome and infantile spasms as synonyms. Neurodevelopmental arrest or regression before the onset of spasms is not required for the diagnosis of WS by the aforementioned Proposal [3]. This epileptic disorder occurs most frequently within 12 months of life, with a peak between 4 and 7 months. However, cases with later onset have been reported [4].

Epileptic spasms are brief contractions (flexions, extensions, or mixed) of the neck, trunk, and upper and lower limbs or head nodding, occurring in clusters, frequently on awakening. They last about 1–2 s and are followed by a tonic phase lasting up to 10 s [3,5]. Their origin is either focal, generalized, or both; thus, they can have a symmetrical or asymmetrical form. Subtle spasms may also occur in the course of this condition. They are easily overlooked and take the form of yawning, gasping, eye deviation, facial grimacing, or focal motor activity. They are associated with hypsarrhythmia [3]. Although the definition of hypsarrhythmia has not been established by the West Delphi Proposal [3], some authors define it as an interictal EEG pattern, which is characterized by continuous, asynchronic, disorganized background of high-amplitude, slow waves and coexisting multifocal spikes, polyspikes, sharp waves, and spike-waves [6]. Hypsarrhythmia is not always present in patients suffering from infantile spasms, and it can be modified/atypical depending on the stage of brain development and physiologic situations, such as sleep/wakefulness, treatment with AED, and etiology [6].

Classifications regarding etiology have been established, including the 1989 International League Against Epilepsy division into two subgroups: symptomatic and cryptogenic [7]. The first one means that etiology is known, or previous signs of brain damage, such as psychomotor retardation, neurologic signs, radiologic signs, or other types of seizures, have been present. The latter one means that signs of previous brain damage have been absent, and etiology has not been identified during medical examinations [7]. Many studies prove that cryptogenic patients have a better response to ACTH treatment, and their neurodevelopmental outcome is also more favorable [5,8,9,10,11,12]. Nowadays, according to the ILAE 2017 classification, etiologies are more precisely divided into structural, infectious, genetic, metabolic, immune, and unknown [13].

ACTH, vigabatrin, and corticosteroids are effective and commonly used in infantile spasms [4,14]. There are also some limited data about other non-standard treatment options, such as topiramate, levetiracetam, zonisamide, sodium valproate, benzodiazepines, pyridoxine, and dietary treatments, which seem to be especially promising for topiramate and ketogenic diet [4,14,15]. On the other hand, using non-standard therapies as a first-line treatment was associated with worse response rate [16,17]. There is also evidence that shorter lead time to effective treatment results in better developmental outcome [9,11].

The aim of the study was to assess the short-term efficacy (during treatment schedule) and long-term outcome of intramuscular 0.02 mg/kg/day ACTH (tetracosactide) depot, used concomitantly with other antiepileptic drugs in patients with infantile spasms who did not achieve seizure cessation or relapse when taking only the AEDs.

## 2. Materials and Methods

### 2.1. Patient’s Characteristics

Fifty children (29 male, 21 female) diagnosed with infantile spasms were included in the study. The age at the onset of epileptic spasms varied between 1 and 11 months, while the mean was 5.2 ± 2.2 months. The age at the onset of ACTH therapy was 8.6 (6–9) months. According to the ILAE classification, 41 (82%) patients could be classified as symptomatic. A detailed presentation of patients with known etiology is shown in Table 1. Other types of seizures prior to the onset of epileptic spasms were present in 17 (34%) patients, and developmental delay was observed during hospitalization in 45 children (90%, the first developmental evaluations were performed at onset in hypsarrhythmic state), microcephaly in 13 (26%), and dysmorphic features in 17 (34%). The family history was relevant in the case of 10 (20%) patients. Ten (20%) patients were born preterm. Abnormalities in MRI were present in 35 cases (70%).

### 2.2. Study Pattern

This study was conducted by a retrospective and prospective analysis of patients hospitalized in the Department of Pediatric Neurology of the Medical University of Silesia in Katowice diagnosed with infantile spasms who were admitted between March 2009 and September 2017. Table 2 shows the onset of epilepsy depending on presence or absence of hypsarrhythmia. Patients who were not treated with ACTH were excluded from the study. ACTH was not the first-line treatment in this population and was used concomitantly with other antiepileptic drugs. AEDs taken by the participants of the study during and before the onset of tetracosactide therapy were noted. Tetracosactide was used at a dose of 0.02 mg/kg/d for two weeks and tapered to zero over the next 4 or 6 weeks—the exact treatment schedule is presented in Section 3 (Table 3). Patients with treatment lasting 6 weeks had the same schedule without the last 2 weeks with one dose per week. The time of ACTH administration was shortened to 5 weeks in one patient because she was qualified for the neurosurgical operation of hypothalamic hamartoma.

Video EEG was taken in all patients. Video EEG and EEG were performed at two-week intervals and after the end of ACTH treatment. Improvement of EEG recording in patients with hypsarrhythmia meant its withdrawal and, in other cases, reduction of paroxysmal changes and their amplitude and the dominance of slow theta and delta waves.

As far as the causes of epilepsy are concerned, patients were categorized according to the 2017 ILAE classification as structural, genetic, infectious, metabolic, immune, and unknown. Some patients could be classified into more than one category. The primary clinical outcome was cessation of spasms, defined as the lack of observed seizures until the 14th day of ACTH treatment, and a seizure-free interval during hospitalization. Patients who achieved cessation of epileptic spasms did not have seizures for 28 consecutive days or more from the last witnessed seizure.

### 2.3. Statistical Analyses

Continuous variables were expressed as the mean ± standard deviation or median (lower quartile-upper quartile), when distribution was not normal. Shapiro–Wilk test and Q-Q plots were used to test normality. Categorical variables were presented as numbers and percentages using Open Office (Apache Open Office 4.1.2 Copyright 2015 The Apache Software Foundation). Continuous data were compared by means of the Mann–Whitney U test. To compare the categorical variables in the short- and long-term outcome, the chi-square test with Yates’s correction and Fisher’s exact probability test were used, respectively. The data were compared using Statistica 12 (Tulusa, OK, USA). Statistical significance was defined as *p* < 0.05.

### 2.4. Treatment Schedule

All patients took at least one AED prior to tetracosactide, and they did not achieve cessation of spasms or had recurrence of epileptic spasms when they started ACTH therapy. The time from diagnosis of epilepsy to administration of the first AED was longer than 30 days in 7 symptomatic patients. The average time lag between the onset of epileptic spasms and the beginning of ACTH therapy was 1.5 (1–3) months. ACTH was used with many other AEDs as add-on therapy. The median number of drugs used prior to tetracosactide was 3.66 ± 1.85 (Table 4). Some patients were undergoing discontinuation of other AEDs when they started ACTH therapy. The following drugs were used during the first two weeks of ACTH therapy:valproate in 41 (82%) patients, vigabatrin in 36 patients, levetiracetam in 9 patients, clobazam in 8 patients, clonazepam in4 patients, phenobarbital in 2 patients, lamotrigine in 2 patients, and topiramate in 2 patients. The mean number of drugs used during ACTH treatment was 2.06.

Twenty-five (50%) patients used the two most common drugs—both vigabatrin and valproic acid at the same time. During the whole period of ACTH therapy, the children were hospitalized.

## 3. Results

Complete cessation of spasms was achieved in 42 cases out of 50 children (84%) withcombined antiepileptic therapy (ACTH and AEDs). Among children who responded to ACTH, 20 were with hypsarrhythmia. EEG improvement was seen in 41 (82%) patients. Improvement of EEG recording in patiets with hypsarrhytmia meant its withdrawal and, in other cases, reduction of paroxysmal changes and their amplitude and the dominance of slow theta and delta waves. A substantial reduction (75%) in the frequency of seizures was present in five patients (10%). Two of them were seizure-free after 2 weeks of treatment but had recurrence of seizures during a tapering-off period. In patients with genetic etiology, therapy was effective in 5/8 (62.5%) patients. The treatment failed in one patient with tuberous sclerosis (TSC), one with a mitochondrial disease, and one with microdeletion 2q24.4. Efficacy in patients with structural etiology other than TSC was 17/20 (85%). In this group, the treatment was effective in 10/11 patients (91%) with HIE, 5/7 (72%) with cerebral malformations, one patient with two possible causes of epilepsy, and one patient with postinfectious changes. In patients with unknown etiology, the therapy was effective in 20 patients (91%), including all patients who could be classified as cryptogenic according to the previous ILAE classification. A total of 80.5% of patients previously classified as symptomatic had cessation of spasms. The differences between cryptogenic and symptomatic patients in the chi-square test were not statistically significant (*p* = 0.345) (using of terms symptomatic and cryptogenic, although not recommended by ILAE, allow for better comparison with previous study). There were also no statistically significant differences between the children with known and unknown etiology (*p* = 0.427). Gender did not have any influence on cessation of seizures (*p* = 0.501).

### 3.1. Adverse Events during ACTH Treatment

There was no case of death during ACTH therapy. The most common adverse events during treatment and taper were infections (respiratory tract, urinary system, gastrointestinal), N = 26 (52%). Most of them were mild and did not have an impact on the epilepsy treatment. Antibiotics were used in 19 (38%) patients. In six (12%) patients, stereotyped dyskinesia (usually of the facial region: oral buccal) was observed. These movement disorders stopped gradually within a month of discontinuation of ACTH treatment. Arterial hypertension was present in five (10%) patients. Hypertension had been diagnosed by cardiologist, who made the decision of temporary use of blood pressure-lowering drugs. In all patients, the blood pressure values returned to normal after completion of the steroid therapy.

Hypokalemia was noted in four (8%) patients.

### 3.2. Follow-Up

Information on the clinical course of 28 patients was obtained during phone follow-up. The study was conducted between June and September 2017. We focused on the presence or absence of seizures (not only IS), drug therapy, and development.

The average follow-up was 37 ± 24.4 months. Table 5 shows the duration of follow-up.

In 17 (60.7%) cases, seizures were still present. Among patients without seizures, six children were classified as cryptogenic, and five children (23%) were recognized as symptomatic (three children were diagnosed with mitochondrial encephalopathy, one with neurofibromatosis type 1, and one with microdeletion 2q24.4). There was not any significant difference related to gender (*p* = 1.0).

Twenty-seven children (97%) were still on anti-epileptic therapy. Most commonly used were valproate, vigabatrin, and levetiracetam in 20, 14, and 12 patients, respectively. Moreover, in 18 cases, valproate was in combination with other drugs: in 10 children, vigabatrin; in 8 cases, levetiracetam; in 3 cases, clobazam; in 3 cases, clonazepam; in 2 children, topiramate; and in 1 case, lacosamide.

Normal or near-normal development was observed in 11 out of 28 children (39%).The clinical outcomes of all 28 cases and the comparison of symptomatic and cryptogenic patients are shown in Table 6.

## 4. Discussion

The mechanism of action of ACTH is still unknown. Treatment with ACTH may reduce neuronal excitability by lowering the production and release of CRH and by directly affecting limbic neurons via melanocortin specific receptors, mainly MC1R and MC3R [1,2,3,4,5,6,7,8,9,10,11,12]. Randomized, controlled hormonal-treatment trials in West syndrome/epileptic spasms are shown in Table 7.

In this study, we present high short-term efficacy of ACTH used concomitantly with other AEDs in a group of patients with IS who did not achieve cessation of seizures or relapsed when taking other AEDs. Monitored adverse events were common (especially infections) but acceptable, and they did not require ACTH discontinuation. Dyskinesia occurred in six (12%) patients. In follow-up, effects were better in cryptogenic patients.

Adverse events in randomized, controlled trials, in which at least one group used hormonal treatment, and were published after 2010 are listed in Table 7.

Using ACTH in polytherapy is an interesting approach, and some studies suggest that it can be more effective than monotherapy in infantile spasms treatment [18,19,20]. A randomized, multicenter, open-label trial provided evidence that hormonal therapy (tetracosactide, prednisolone) and vigabatrin are more effective than hormonal therapy alone during four weeks of observation [18]. A randomized, open-label study from China also suggested that ACTH in polytherapy with MgSO4 has a better effect than ACTH monotherapy in 24 weeks of follow-up [19]. Prospective study at Severance Children’s Hospital also demonstrated the potential of a treatment protocol using polytherapy(vigabatrin and prednisolone) for West syndrome [20]. Other studies also showed that initial polytherapy with ACTH and other AED or topiramate followed by low-dose ACTH are effective and well-tolerated options in IS [21,22,23]; however, it is worth noting that the randomized, open-label, controlled trial denied the effectiveness of combination of hormonal therapy(prednisone) and moderate dose of to piramate [24].

In our study, short-term efficacy seems to be high in comparison with the results from randomized studies from the last ten years (Table 6) [18,19,24,25,26,27,28,29,30,31,32,33,34].

The number of patients with cessation of IS in our study is very similar to the groups using hormonal treatments in combination with other drugs [18,19,20,35]. Relatively good short-term efficacy of the add-on ACTH polytherapy in our study might be associated with cumulative effects of different drugs suggested by the previously mentioned studies, especially vigabatrin, which was taken by most patients [18,19,20,35]. Valproate was also commonly used in our study and might have had an important role in the reached outcome, but there are limited data about its efficacy in IS [14,36]. In addition, clobazam used in our study might be a potentially effective adjunctive drug for West syndrome [37]. It is worth mentioning that similar results were reached in some old studies in the case of high-dose ACTH monotherapy and combination of low-dose ACTH and pyridoxine [23,38]. According to some authors, increasing the dose of ACTH does not necessarily result in better seizures control [39].

However, on the other hand, we would not favor polytherapy because of the risk of serious adverse effects (e.g., potential hepatotoxicity in infancy). There is also no proven evidence of effectiveness of polyteraphy in infantile spasms. Based on observation, we would like to stress that it is worthwhile to start ACTH even after refractory spasms and relapses and even after a long delay.

Monitored adverse events reported in this study were relatively mild, and they did not require discontinuation of treatment (Table 8).

**Table 7:** Randomized, controlled hormonal-treatment trials published after 2010.

Study	Therapy	No. of Patients	Etiology	Cessation of Seizures Rate (%)
Zou 2010 [19]	ACTH 25 U/d and MgSO_4_ 0.25 g/kg/d 3 weeks	19	Cryp. 3 (16%) Symp. 16 (84%)	1st week, 42% 2nd week, 53% 4th week, 63% 8th week, 79% 12th week, 74% 24th week, 63%
	ACTH 25 U/d 3 weeks	19	Cryp. 2 (11%) Symp. 17 (89%)	1st week 5% 2nd week 21% 4th week 42% 8th week 53% 12th week, 47% 24th week, 53%
Chellamuthu et al., 2014 [28]	Oral prednisolone 2 mg/kg/d	32	Known, 27 (84%) Unknown, 5 (16%)	14 days, 25%
	Oral prednisolone 4 mg/kg/d	31	Known 26 (84%) Unknown 5 (16%)	14 days, 51.6%
Wanigasinghe 2015 [29] Wanigashingasighe 2017 [30]	Corticotropin 0.5–0.75 mg (40 IU)/2 d 2 weeks and taper	49	Known 68% Unknown 24% Incomplete evaluation 7%	14 days, 37% 42 days, 41% 3 months, 39% 6 months, 45% 12 months 41%
	Oral prednisolone 40–60 mg/d 2 weeks and taper	48		14 days, 58% 42 days, 67% 3 months, 65% 6 months, 58% 12 months, 56%
O’Callaghan et al., 2017 [18] O’Callaghan et al., 2018 [31]	Prednisolone 40–60 mg/d or tetracosactide 0.5–0.75 mg (40 IU)/2 d 2 weeks and taper Vigabatrin 50–150 mg/kg/d 3 months and taper	186	Known, 219 (58%) Unknown, 158 (42%)	14 days 89% 42 days, 72% At age 18–19 months, 70%
	Prednisolone 40–60 mg/d or tetracosactide 0.5–0.75 mg (40 IU)/d 2 weeks and taper	191		14 days 69% 42 days, 57% At age 18–19 months, 71%
Kunnanayaka V. 2018 [32]	Prednisolone 4 mg/kg/d	32	Known, 27 (84%) Unknown, 5 (16%)	14 days 37.5% 1 month, 25%
	Prednisolone 4 mg/kg/d and 30 mg/kg/d pyridoxine	30	Known, 26 (86%) Unknown, 4 (14%)	14 days, 37% 1 month, 33%
ZhaoshiYi et al., 2019 [24]	Prednisone 4 × 10 mg/d ^a^	39	Known 25 (64.1%) Unknown 14 (35.9%)	14 days 71.8% 120 days 61.5%
	Prednisone 4 × 10 mg/ ^a^ and TPM gradually titrated from 1 mg to 5 mg in 14 day/kg/d ^b^	38	Known 23 (60.5%) Unknown 15 (39.5%)	14 days 76.3% 120 days 50.0%
Angappan et al., 2019 [33]	Tetracosactide 30–60 IU/d, the dose increasing every 2–3 days by 10 IU and taper	15	Structural 12 (80%) Non-structural 3 (10%)	2 weeks till 6 weeks 40%
	Oral zonisamide 4–25 mg/kg/day	15	Structural 100%	2 weeks till 6 weeks 27%
Gowda et al., 2019 [34]	ACTH 100 IU/body surface area/d	18	Symp 14 (77.77%) Idiop 1 (5.55%) Cryp. 3 (16.66%)	14 days 50% 28 days 61.11%
	PRDL 4 mg/kg/d for 2 weeks, then tapered over 3–4 weeks)	16	Symp 13 (81.25%) Idiop 0 (0%) Cryp. 3 (18.75%)	14 days 33.33% 28 days 40%
Dressler et al., 2019 [25]	ACTH 150 IU/m^2^ for 2 weeks and taper	16	Known 11 (69%) Unknown 5 (31%)	24 month 44%
	Ketogenic diet	16	Known 7 (44%) Unknown 8 (66%)	24 month 38%
Fayyazzi et al., 2020 [26]	ACTH 0.1 mg/d	16	Known 25 (78%) Structural 17 (53%) Unknown 7 (22%)	^c^
	ACTH 0.25 mg/d ^d^	16		^c^
Imannezhad et al., 2020 [27]	ACTH 5 × 2–3 IU/kg/d (max: 100 IU)	25	N.A.	14 days 69.2%
	Prednisolone 8 mg/kg/d (max: 60 mg) ^e^	26		14 days 76%

^a^ If spasm still occurred on day 7, the dose was changed; it reached 15 mg once and was kept at this level for the next 14 days, four times each day. Whether the spasms hadstopped or not, after these 14 days, the dose of prednisone was decreased every week until completing a 49-day or 56-day course (e.g., 40 mg one time a day for a week or 30 mg one time a day for a week, 20 mg one time a day for a week, 10 mg one time a day for a week, 5 mg one time a day for a week, and finally, 5 mg every two days for a week). ^b^ Additionally, the combination therapy group used TPM at an initial dose of 1 mg/kg/day twice a day, then gradually increased it to 3 mg/kg/day on day 7 and 5 mg/kg /day on day 14. ^c^ No further medications were needed at the end of the therapeutic protocol since symptoms, such as convulsions and spasm, were fully controlled in 18.7% of patients. Apart from ACTH, another medicine was given to 5.37% of patients to fully control the convulsions. Using another drug helped 25% of patients with controlling, to some extent, theoccurring convulsions and spasms. The mentioned symptoms were resistant to treatment in 3.9% of patients despite concomitant treatment with ACTH and several other drugs. There was no significant change in any of the four levels defined for controlling spasms and convulsions. ^d^ The method of ACTH administration: 1st week, one time a day; 2nd week, once every second day; 3rd week, two times a week; 4th week, one weekly; and 5th to 8th week, once every two weeks. Increase daily dose if the patient was not responsive after two weeks; 14 days of therapy, and then, the dose was tapered off over the remaining two weeks. ^e^ A dose of 8 mg/kg/day of prednisolone (max: 60 mg) was administrated to patients who belonged to the corticosteroid group. It was used for three weeks, divided into three doses. Later, the dose was decreased in responders. Two weeks later, non-responders were given 2–3 U/kg/day of ACTH (max: 100 U) for five days. If the patients did not show signs of improvement within two weeks of treatment, they were planned to receive intramuscular biologic ACTH immediately after the failure of corticosteroid therapy. The patients received five daily doses of 2–3 IU/kg of ACTH (max: 100 IU), and the treatment was then changed to the oral corticosteroid. In the second group (25 patients), patients first received five daily doses of 2–3 IU/kg of ACTH.

**Table 8:** Adverse events in randomized, controlled trials, in which at least one group used hormonal treatment, and were published after 2010, which were found in bases PubMed, Google Scholar, and Embase. Studies about including patients without IS are not presented in the table.

Study	Therapy 1		Therapy 2	
	Adverse Events	No (%)	Adverse Events	No (%)
Zou 2010 [19]	ACTH 25 U/d and MgSO4 0.25 g/kg/d 3 weeks (19) *		ACTH 25 U/d 3 weeks (19) *	
	Pyrexia	3 (15.8)	Pyrexia	3 (15.8)
	Upper respiratory tract infection	3 (15.8)	Upper respiratory tract infection	3 (15.8)
	Diarrhea	2 (10.5)	Diarrhea	2 (10.5)
	Anorexia	1 (5.3)	Anorexia	1 (5.3)
	Vomiting	0 (0)	Vomiting	1 (5.3)
	Hypertension	2 (10.5)	Hypertension	0 (0)
	Insomnia	2 (10.5)	Insomnia	0 (0)
	Irritability	2 (10.5)	Irritability	0 (0)
	Decreased heart rate, prolonged PR interval	1 (5.3)	Decreased heart rate, prolonged PR interval	0 (0)
Chellamuth et al., 2014 [28]	Oral prednisolone 2 mg/kg/d (32) *		Oral prednisolone 4 mg/kg/d (31) *	
	Weight gain	4 (12.5)	Weight gain	9 (29)
	Hypertension	1 (3.12)	Hypertension	0 (0)
	Cushingoid facies	7 (22.6)	Cushingoid facies	13 (41.9)
	Infections	6 (18.7)	Infections	9 (29)
	Irritability	3 (9.4)	Irritability	2 (6.5)
	Increased appetite	2 (6.3)	Increased appetite	4 (12.9)
	Glycosuria	0 (0)	Glycosuria	0 (0)
	Others	2 (6.3)	Others	1 (3)
Wanigasinghe 2015 [29] Wanigassighe 2017 [30]	Oral prednisolone 40–60 mg/d 2 weeks and taper (48) *		Tetracosactide 0.5–0.75 mg (40 IU)/2 d 2 weeks and taper (49) *	
	Increased appetite	28 (73.7)	Increased appetite	19 (43.2)
	Weight gain	19 (50)	Weight gain	14 (31.8)
	Frequent crying spells	16 (42.1)	Frequent crying spells	11 (25)
	Drowsiness	4 (10.5)	Drowsiness	7 (15.9)
	Cushingoid features	8 (21.1)	Cushingoid features	9 (20.5)
	Insomnia	3 (7.9)	Insomnia	2 (4.5)
	Lethargy	2 (5.3)	Lethargy	2 (4.5)
	Reduction in social behavior	2 (5.3)	Reduction in social behavior	1 (2.3)
	Abdominal distension	8 (21.1)	Abdominal distension	0 (0)
	Hypertension	1 (2.6)	Hypertension	1 (2.3)
	Increased susceptibility to infection	0 (0)	Increased susceptibility to infection	1 (2.3)
	Irritability	8 (21.1)	Irritability	5 (11.4)
	Nausea	1 (2.6)	Nausea	1 (2.3)
	Vomiting	2 (5.3)	Vomiting	1 (2.3)
	Diarrhea	2 (5.3)	Diarrhea	3 (6.8)
	Dyspepsia	2 (5.3)	Dyspepsia	2 (4.5)
	Electrolyte imbalances	2 (5.3)	Electrolyte imbalances	0 (0)
O’Callaghan et al., 2017 [18] O’Callaghan et al., 2018 [31]	Prednisolone 40–60 mg/d or tetracosactide 0.5–0.75 mg (40 IU)/d 2 weeks and taper (191) *		Prednisolone 40–60 mg/d or tetracosactide 0.5–0.75 mg (40 IU)/2 d 2 weeks and taper Vigabatrin 50–150 mg/kg/d 3 months and taper (186) *	
	Allergic rash or anaphylaxis	1 (1)	Allergic rash or anaphylaxis	0 (0)
	Drowsiness	3 (2)	Drowsiness	45 (24)
	Endocrine or metabolic disturbance	2 (1)	Endocrine or metabolic disturbance	1 (1)
	Fluid or electrolyte disturbance	23 (12)	Fluid or electrolyte disturbance	12 (6)
	Gastrointestinal upset	26 (14)	Gastrointestinal upset	23 (12)
	Hypertonia	9 (5)	Hypertonia	3 (2)
	Hypotonia	8 (4)	Hypotonia	7 (4)
	Immunosuppression	3 (2)	Immunosuppression	3 (2)
	Increased appetite	51 (27)	Increased appetite	35 (19)
	Infection	19 (10)	Infection	14 (8)
	Irritability	75 (39)	Irritability	61 (33)
	Neuropsychiatric (disturbed sleep)	35 (18)	Neuropsychiatric (disturbed sleep)	29 (16)
	Varicella zoster (chicken pox)	4 (2)	Varicella zoster (chicken pox)	2 (1)
	Weight gain	34 (18)	Weight gain	24 (13)
	Abnormal eye movements	0 (0)	Abnormal eye movements	1 (1)
	Blood disorder (high platelet count)	0 (0)	Blood disorder (high platelet count)	1 (1)
	Bradycardia	0 (0)	Bradycardia	1 (1)
	Abnormal breathing pattern	1 (1)	Abnormal breathing pattern	0 (0)
	High MRI signal in basal ganglia	1 (1)	High MRI signal in basal ganglia	2 (1)
	Hypoxia	1 (1)	Hypoxia	0 (0)
	Movement disorder	2 (1)	Movement disorder	14 (8)
	Not focusing (vision)	0 (0)	Not focusing (vision)	1 (1)
	Obstructive cardiac hypertrophy	1 (1)	Obstructive cardiac hypertrophy	0 (0)
	Pallor	1 (1)	Pallor	0 (0)
	Squinting	1 (1)	Squinting	0 (0)
	Sweating	1 (1)	Sweating	1 (1)
	Tachypnoea	1 (1)	Tachypnoea	0 (0)
Kunnanayaka V 2018 [32]	Prednisolone 4 mg/kg/d (32) *		Prednisolone 4 mg/kg/d and 30 mg/kg/d pyridoxine (30) *	
	Increased appetite	25 (78)	Increased appetite	24 (75)
	Irritability	19 (59)	Irritability	12 (40)
	Excessive daytime sleepiness	21 (66)	Excessive daytime sleepiness	20 (67)
	Cushingoid facies	5 (16)	Cushingoid facies	4 (13)
	Oral ulcers	4 (13)	Oral ulcers	6 (20)
	Weight gain	5 (16)	Weight gain	4 (13)
ZhaoshiYi et al., 2019 [24]	Prednisone 4 × 10 mg/d (39) *		Prednisone 4 × 10 mg/and TPM gradually titrated from 1 mg to 5 mg in 14 day/kg/d (38) *	
	Cushing’s symptoms	34 (87.2%)	Cushing’s symptoms	32 (84.2)
	Increased appetite	35 (89.7)	Increased appetite	29 (76.3)
	Irritability	18 (46.2)	Irritability	12 (31.6)
	Drowsiness	8 (20.5)	Drowsiness	7 (18.4)
	Intercurrent infection	11 (28.2)	Intercurrent infection	12 (31.6)
	Hypertension	1 (2.6)	Hypertension	0 (0.0)
	Sleep disturbance	9 (23.1)	Sleep disturbance	7 (18.4)
Angappan et al., 2019 [33]	Tetracosactide 30–60 IU/d, the dose increasing every 2–3 days by 10 IU and taper (15) *		Oral zonisamide 4–25 mg/kg/day (15) *	
	Hypertension	14 (93.3)	Lethargy	8
	Weight gain	5 (33.3)	Irritability	5 (33.3)
	Cushingoid faces	3 (20)	Gastroenteritis	2 (13.3)
	Irritability	3 (20)	Dryness of skin and mouth	1 (6.7)
	Infection	2 (13.3)	Loss of appetite	1 (6.7)
	Hyperpigmentation	1 (6.7)	Metabolic acidosis	1 (6.7)
Gowda et al., 2019 [34]	ACTH 100 IU/body surface area/d (18) *		PRDL 4 mg/kg/d for 2 weeks, then tapered over 3–4 weeks) (16) *	
	Side effects in general	3 (16.6)	Side effects in general	3 (20)
Dressler et al., 2019 [25]	ACTH 150 IU/m2 a 2 weeks and taper (48) *		Ketogenic diet (53) *	
	Adverse effects overall	45 (94)	Adverse effects overall	42 (79)
	Needing acute intervention	45 (94)	Needing acute intervention	16 (30)
	Hypertonia	41 (85)	High triglycerides	16 (30)
	Potassium (intravenous)	19 (40)	Obstipation	14
	Cushing syndrome	17 (35)	Ketones > 5 mmol L^−1^	13
	Cardiac hypertrophy	16 (33)	Solid food refusal	9
	Leukocytosis	16 (33)	Liquids (intravenous)	7
	Infections	14 (29)	Infections	6 (11)
	Hyperexcitability	12 (25)	Diarrhea	6 (11)
	Acne	12 (25)	High cholesterol	5 (9)
	Weight gain	11 (23)	Growth deficit	5 (9)
	Drowsiness	8 (17)	Cholecystolithiasis	5 (9)
	Edema	8 (17)	Tiredness at start	3 (6)
	-	-	Hypoglycemia	3 (6)
	-	-	Carnitine deficiency	3 (6)
	-	-	Weight loss	3 (6)
	-	-	Refusal of KD liquids	3 (6)
	-	-	Weight gain	1 (2)
Fayyazzi et al., 2020 [26]	ACTH 0.1 mg/d (16) *		ACTH 0.25 mg/d (16) *	
	N.A	N.A	N.A	N.A
Imannezhad et al. 2020 [27]	ACTH 5× 2-3IU/kg/d(max: 100 IU) (25) *		Prednisolone 8 mg/kg/d(max: 60 mg) (26) *	
	N.A	N.A	N.A	N.A

* Number of patient

Dyskinesia is a newly reported side effect of hormonal treatment. In this study, six (12%) patients had a dyskinesia similar to that previously reported [40,41]. Many patients in this study took vigabatrin, and movement disorders might be also potentially associated with vigabatrin [42,43]. In ICISS, the group treated with the combination of hormonal therapy and vigabatrin had higher rate of movement disorders than the group taking hormonal therapy alone [18]. In ten investigated patients enrolled in ICISS, eight of them had movement disorders after initiation of vigabatrin, but they resolved after vigabatrin withdrawal only in two patients. Seven of them also took hormonal treatment during the onset of the movement disorders [42]. Due to the ICISS report, mentioned movement disorders were not linked to vigabatrin, and they were likely caused by an underlying neurological disease [18].

In our study, the number of patients with a developmental delay and seizures in a long-term perspective was significantly lower in cryptogenic patients, which is consistent with previous studies [8,9,10,11,12,31]. In total, 39% of them were seizure free, and among them, all were cryptogenic patients (100%), and 22.7% were symptomatic patients. This result resembles long-term outcome in other studies; however, some treatment options used in the studies presented in the Table 6 resulted in better long-term outcome, which can be caused by differences in methodology and time of observation [11,19,25,26,27,29,30,33,34,44]. Cryptogenic patients seem to have slightly better and symptomatic slightly worse seizure outcome than patients from UKISS and the observational multicenter Belgian study by Mert et al. [10,11,12]. Development was normal or near normal in 39% of patients—83% cryptogenic and 27% symptomatic patients. According to the meta-analysis and systematic review published in 2015, 54.3% of cryptogenic and 12.5% of symptomatic patients with IS had normal or near-normal developmental outcome [9]. In the context of these data, the developmental outcome in our study seems to be good. On the other hand, there is a possibility that assessment of development was stricter in other studies. Shorter time to treatment results in better developmental outcome, especially in cryptogenic patients [8,9,31,45]. Good developmental outcome among cryptogenic patients in our study might be also associated with short lead time to treatment in this group of patients.

It should be mentioned that according to the latest meta-analysis, high-dose prednisolone is not inferior to adrenocorticotrophic hormone [46,47]; indeed, ACTH is less cost-effective, defined as probability of clinical spasm resolution 14 days after treatment initiation, than oral prednisolone (in the United States) [48].

Our study has some weakness and limitations. The first is a partially retrospective character. Others are an inaccessibility of almost half of patients for follow-up and differences in time of follow-up for particular patients. The follow-up method, phone interview, might be less accurate than direct clinical assessment. The study did not assess some ACTH adverse events. This study is not a clinical trial but only a description of experience from our clinic. There was not a control group, so it does not allow for making an accurate comparison for different used drugs or approaches in infantile spasms treatment; it only assesses effectiveness of the add-on ACTH therapy in this group of patients.

## 5. Conclusions

ACTH used concomitantly with other AED is a highly effective treatment with acceptable side effects. In accordance with other studies, the long-term outcome was better throughout cryptogenic patients. Randomized, controlled, clinical trials with long-term follow-up are needed to compare effectiveness of ACTH in polytherapy and monotherapy. Dyskinesias as a potential side effect observed in our study group should be investigated in future studies.

## Figures and Tables

**Table 1:** Epilepsy etiology.

Etiology		
Structural	Genetic	Metabolic
- 15 (50%) hypoxic-ischemic encephalopathy - 8 (16%) cerebral malformation - 1 (2%) hypothalamic hamartoma and cerebral malformation - 1 (2%) postinfectious changes	- 4 (8%) tuberous sclerosis - 2 (4%) *CDKL5* mutation - 1 (2%) neurofibromatosis type, 1 - 1 (2%) microdeletion 2q24.4 - 1 (2%) *SPTAN* mutation - 1 (2%) *CHD2* mutation - 1 (2%) *ARX1* mutation - 1 (2%) *STXBP1* mutation	- 3 (6%) mitochondrial diseases - 1 (2%) nonketotic hyperglycinemia

**Table 2:** Epilepsy onset: 2 days–11 months.

Mean Age of the Patients at the First Seizures (months)	
Patients with hypsarrhythmia	5.5 SD = 2.3
Patients without hypsarrhythmia	3.3 SD = 2.0
Study group	4.6 SD = 2.1

**Table 3:** Schedule of treatment shows frequency of 0.02 mg/kg/d ACTH administration in particular weeks of treatment.

Weeks of Treatment	Frequency of Administration
1–2	Every day
3–4	Every second day
5–6	2 times in a week
7–8	Once in a week

**Table 4:** Antiepileptic drugs used before the onset of ACTH.

Antiepileptic Drugs	Patients with Hypsarrhythmia	Patients without Hypsarrhythmia	Study Group
Vigabatrin	N = 22 (92%)	N = 14 (82%)	N = 36 (88%)
Valproic acid	N = 22 (92%)	N = 15 (88%)	N = 37 (90%)
Levetiracetam	N = 5 (21%)	N = 9 (52%)	N = 14 (34%)
Clobazam	N = 5 (21%)	N = 8 (47%)	N = 13 (31%)
Phenobarbital	N = 3 (13%)	N = 6 (35%)	N = 9 (22%)
Clonazepam	N = 3 (13%)	N = 3 (18%)	N = 6 (15%)
Carbamazepine	N = 0	N = 3 (18%)	N = 3 (7%)
Lamotrigine	N = 1 (4%)	N = 2 (12%)	N = 3 (7%)
Phenytoin	N = 0	N = 2 (12%)	N = 2 (5%)
Topiramate	N = 2 (8%)	N = 1 (6%)	N = 2 (5%)
Acetazolamide	N = 1 (4%)	N = 1 (6%)	N = 1 (2%)
Nitrazepam	N = 0	N = 1 (6%)	N = 1 (2%)

**Table 5:** Duration of follow-up.

Length of Follow-Up Study (Months)	<13	13–24	25–36	37–48	49–60	61–72	>72
Number of patients (%)	5 (18)	4 (14)	7 (25)	3 (11)	2 (7)	3 (11)	4 (14)

**Table 6:** Differences in follow-up between cryptogenic and symptomatic patients.

	Cryptogenic Patients (%)	Symptomatic Patients (%)	*p*-Value
Seizures			0.001
absent	6 (100)	5 (23)	
present:	0	17 (77)	
more than 2 per month	0	11 (50)	
less than 2 per month	0	6 (27)	
AED			0.078
absent	0	1 (5)	
monotherapy	2 (33)	2 (9)	
2 drugs	4 (67)	9 (39)	
3 or more	0	10 (43)	
Mean	1.7 SD = 0.5	2.4 SD = 1.0	
Development:			0.022
unfavorable	1 (17)	16 (73)	
favorable	5 (83)	6 (27)	

## Data Availability

The data analyzed during the current study are available from the corresponding author on reasonable request.
